# Supplementary material for: Conditional chemoconnectomics (cCCTomics) as a strategy for efficient and conditional targeting of chemical transmission
Source: eLife. 2024 Apr 30;12:RP91927. doi: 10.7554/eLife.91927 (PMC11060718; doi:10.7554/eLife.91927)
Supplement: Figure 3—source data 3. [file elife-91927-fig3-data3.zip › Figure 3 source data3 part 2.zip!/Figure 3-Source data 3-part2/1052-13-CRISPResso_Report_ejedac2.zip!/CRISPResso_on_1052_14/9.Alleles_frequency_table_around_sgRNA_agtggacaaactggtacatg.pdf]

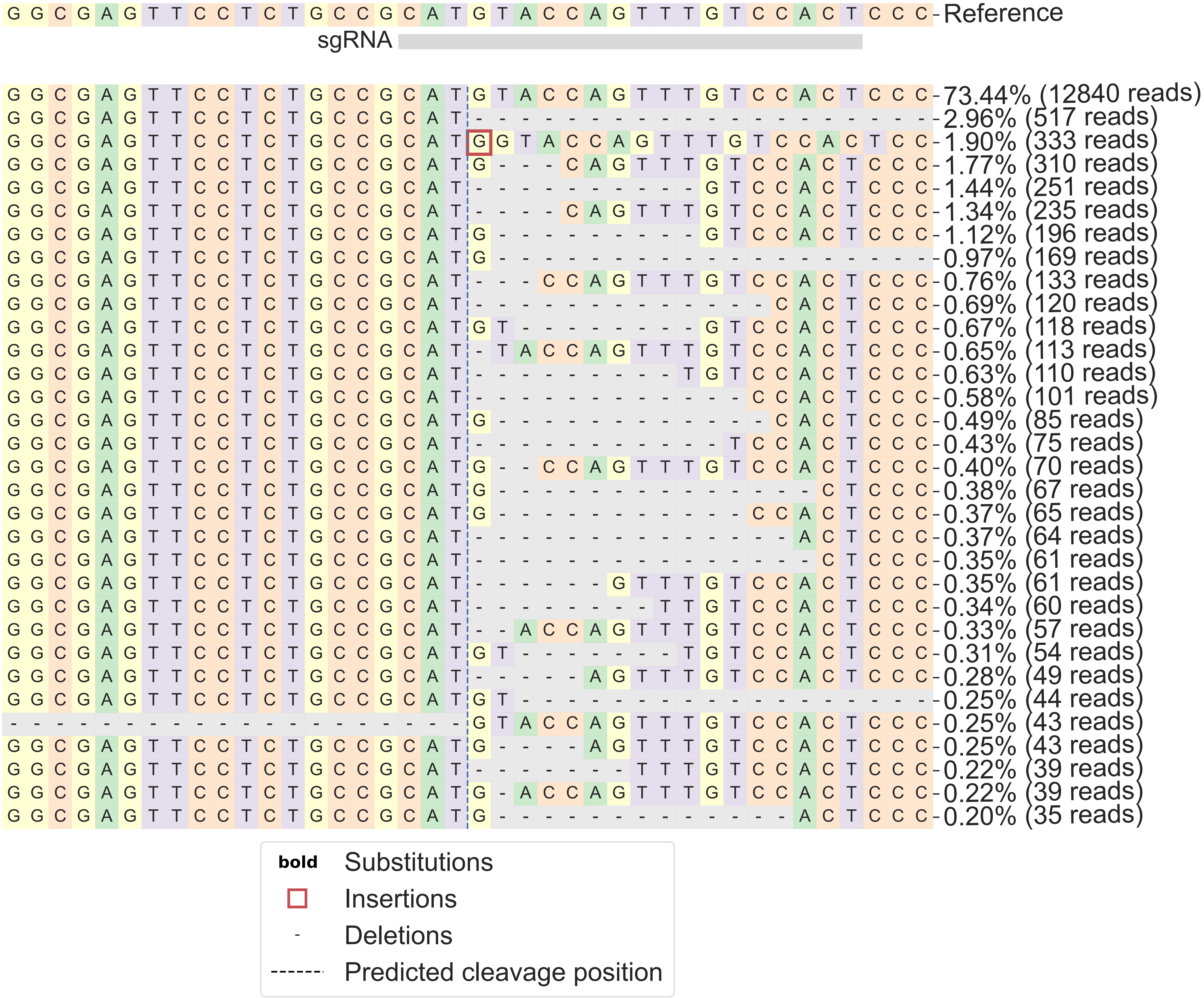

G G C G A G T T C C T C T G C C G C A T G T A C C A G T T T G T C C A C T C C C
Reference
sgRNA
73.44% (12840 reads)
2.96% (517 reads)
1.90% (333 reads)
1.77% (310 reads)
1.44% (251 reads)
1.34% (235 reads)
1.12% (196 reads)
0.97% (169 reads)
0.76% (133 reads)
0.69% (120 reads)
0.67% (118 reads)
0.65% (113 reads)
0.63% (110 reads)
0.58% (101 reads)
0.49% (85 reads)
0.43% (75 reads)
0.40% (70 reads)
0.38% (67 reads)
0.37% (65 reads)
0.37% (64 reads)
0.35% (61 reads)
0.35% (61 reads)
0.34% (60 reads)
0.33% (57 reads)
0.31% (54 reads)
0.28% (49 reads)
0.25% (44 reads)
0.25% (43 reads)
0.25% (43 reads)
0.22% (39 reads)
0.22% (39 reads)
0.20% (35 reads)
bold Substitutions
Insertions
Deletions
Predicted cleavage position
